# Mortality and Function After Hip Fracture or Pneumonia in People With and Without Dementia

**DOI:** 10.1111/jgs.19354

**Published:** 2025-01-15

**Authors:** Lauren J. Hunt, R. Sean Morrison, Siqi Gan, Edie Espejo, W. John Boscardin, Rebecca Rodin, Katherine A. Ornstein, Alexander K. Smith

**Affiliations:** 1 Philip R. Lee Institute for Health Policy Studies, University of California, San Francisco, California, USA; 2 Department of Epidemiology and Biostatistics, University of California, San Francisco, California, USA; 3 Global Brain Health Institute, University of California, San Francisco, California, USA; 4 Brookdale Department of Geriatrics and Palliative Medicine, Icahn School of Medicine, New York, New York, USA; 5 James J. Peters VA, Bronx, New York, USA; 6 Northern California Institute for Research and Education, San Francisco, California, USA; 7 Division of Geriatrics, University of California, San Francisco, California, USA; 8 School of Nursing, Johns Hopkins University, Baltimore, Maryland, USA

**Keywords:** acute events, dementia, function, mortality

## Abstract

**Background:**

The extent to which disruptive surgical or medical events impact mortality and function is critical for anticipatory planning and informing goal-aligned care.

**Methods:**

Using Health and Retirement Study data (2008–2018), we employed propensity score matching to compare the impact of hospitalization for hip fracture (a surgical event) or pneumonia (a medical event) among people with dementia to two groups: (1) people with dementia who did not experience these events; and (2) people without dementia who experienced an event. Dementia status was determined using validated cognitive assessments (Hurd method); hip fracture and pneumonia were identified from Medicare claims. Outcomes were 1-year mortality and function, defined as a summary score of requiring assistance with 6 ADL’s and 5 IADL’s, with higher scores indicating better function.

**Results:**

Among people with dementia, predicted 1-year mortality was higher among those with hip fracture (35.4%) versus those without hip fracture (14.8%), with similar patterns for pneumonia (49.6% vs. 13.0%). Among people with dementia, function declined abruptly at time of hip fracture (−2.09 [95% CI −2.94, −1.25]) and continued to decline after (−0.48 [95% CI −0.87, −0.09]). There were similar patterns for pneumonia (drop at time of pneumonia of −1.49 [95% CI −2.0, −0.97] and after −0.05 [95% CI, −0.29, 0.19]). Compared to people without dementia with hip fracture, people with dementia had higher 1-year mortality (35.4%) versus people without dementia (24%), with similar patterns for pneumonia (49.6% vs. 39.7%). Function stabilized for people without dementia after hip fracture (−0.03, 95% CI −0.22, 0.16), which was significantly different than people without dementia (*p* < 0.0001). Function improved for people without dementia after pneumonia (0.13, 95% CI 0.03, 0.24), but was not statistically different than for people with dementia (*p* = 0.17).

**Conclusion:**

Disruptive events such as hip fracture or pneumonia substantially alter the clinical trajectories of people with dementia.

## 1 | Introduction

Clinicians may intuitively recognize that hospitalization for a hip fracture or pneumonia represents a disruptive event among community-dwelling people with dementia, and prior research from small, short-term studies of nursing home residents with advanced dementia or hospital-based samples provides limited support for this clinical intuition [1–5]. However, the extent of the impact of disruptive surgical and medical events among community-dwelling people with dementia on long-term mortality and functional trajectories is largely unknown. Confirming this clinical intuition and understanding the extent to which disruptive events such as hip fracture or pneumonia impact important patient-centered outcomes such as mortality and functional decline for community-dwelling people with dementia is critical to creating appropriate treatment plans, providing anticipatory guidance to patients and families, and informing health system needs.

Over two-thirds of people with dementia reside in private residences and other community-based settings [6]. Thus, understanding the impact of these events in community-dwelling samples—and how that compares to people without dementia—is essential to advancing our knowledge surrounding the experiences and needs of the growing dementia population [7]. The existing literature in nursing home and hospital-based samples clearly demonstrate that disruptive events such as hip fracture are associated with poor outcomes for people with dementia in the event’s immediate aftermath. Previous studies show that over 50% of people with advanced dementia die within 6-months of hip fracture or hospitalization for pneumonia and functional decline following events is profound and nearly universal [1–5]. It is unknown whether similar patterns occur for community-dwelling populations of people with dementia and how trajectories progress beyond 6-months to 1-year. We also know that these events occur more frequently in people with dementia compared to those without dementia and a relatively robust body of literature have shown that people with dementia have worse outcomes following these events than people without dementia [8, 9]. However, previous studies comparing people with dementia to those without dementia are similarly limited by their short follow-up periods, lack of data on trajectories prior to the event, and small sample sizes [10–13].

Leveraging a longitudinal cohort from the population-based Health and Retirement Study, we sought to estimate the impact of a disruptive surgical (hospitalization for hip fracture) or medical (hospitalization for pneumonia) event on 1-year mortality and functional trajectories in people with and without dementia.

## 2 | Methods

### 2.1 | Study Design and Sample

The Health and Retirement Study (HRS) is a longitudinal, nationally-representative study designed to examine changes in health and wealth as people age [14]. Begun in 1992, core interviews are conducted every 2 years with replenishment cohorts added every 6 years. Proxy respondents, generally the participant’s next of kin, are interviewed for participants unable to complete an interview and after the participant’s death (exit interview). Response rates range from 85% to 90%.

We included participants interviewed between 2008 and 2018 who were 65 years or older and community-residing (not nursing home residents) at the time of their first eligible interview (*n* = 15,998). We excluded individuals who did not consent to have their HRS surveys linked to Medicare (*n* = 1755), individuals who lacked continuous part A throughout the study period (*n* = 53), individuals with missing dementia status in the follow-up period (*n* = 157), and individuals whose first interview occurred after 2018 (the most recent year of HRS Medicare data available) (*n* = 52) for a final analytic cohort of *n* = 13,981, including 11,502 who never developed dementia in the study period and 2479 who did.

Within the analytic cohort, we identified cases of hospitalization for hip fracture or pneumonia among unique participants (hereafter we reference as hip fracture or pneumonia with the understanding that this represents a hospitalization for these conditions). At each wave, we matched participants with hip fracture (cases) to those without hip fracture (controls) and those with pneumonia (cases) to those without pneumonia (control). To match cases and controls, we calculated propensity scores with the covariates age, sex, dementia status, number of comorbidities, and education level and then applied propensity score matching to match participants, with exact matching required for sex, HRS wave, and dementia status.

For the cases, the date of the hip fracture or pneumonia was set as “time zero.” For controls who did not have hip fracture or pneumonia, we calculated a synthetic hip fracture or pneumonia date based on the interval start date and the matched case hip fracture or pneumonia date (Figure S1). Participants who experienced hip fracture or pneumonia in a later wave could be used as controls in earlier waves, but their function scores were removed after the event occurred (for function analyses only). Most cases (648/655 hip fracture and 2786/2814 pneumonia) were successfully matched. The approach of matching participants at a real or synthetic event time, or “time zero” improves causal inference with observational data by emulating a randomized trial start date [15, 16]. This approach potentially provides less biased estimates than using other time-varying covariate survival analysis approaches that do not use matching [17].

The main focus of our analysis was to compare the impact of hip fracture or pneumonia among people with dementia to two groups: (1) people with dementia who did not experience hip fracture or pneumonia; and (2) people without dementia who experienced hip fracture or pneumonia. For the first comparison, we used the cohort of participants with dementia with and without hip fracture or pneumonia who were matched as described above at real or synthetic event time. For the second comparison, we compared outcomes between people with and without dementia with hip fracture or pneumonia, adjusted for covariates. We included the group with no dementia and no hip fracture or pneumonia in analyses as a baseline comparison. This innovative approach of comparing pre- and post-trajectories for both people with and without dementia and with and without hip fracture or pneumonia allows for a more comprehensive understanding of the impact of these events for people with dementia compared to prior “incident cohort” studies (e.g., cohort begins at the time of hip fracture).

### 2.2 | Measures

While there are numerous examples of medical or surgical events that could be considered disruptive (e.g., heart attack and stroke), we chose hospitalization for hip fracture or pneumonia as exemplar events as we have done in previous work [9]. Hip fracture and pneumonia were chosen because they occur relatively frequently, are associated with poor outcomes in older adults in prior literature, and they were measurable in the Health and Retirement Study-Medicare-linked dataset [18–20].

#### Hip Fracture:

We identified cases of hospitalization for hip fracture and pneumonia in the MedPAR file, which includes data on inpatient admissions. Hip fractures were identified using ICD-9 codes prior to 2015 and ICD-10 codes thereafter based on prior algorithms (Methods S1) [21, 22]. Claims could appear in any position for ICD-9 and in the first or second position for ICD-10. To ensure we identified distinct events, unique events had to be at least 100 days apart. The vast majority (90%–95%) of hip fractures are captured using the inpatient diagnostic claims alone [21, 22]. We opted not to include outpatient claims because we wanted to identify hip fractures requiring hospitalization.

#### Pneumonia:

We opted to include only pneumonia that required hospitalization because we wanted to create consistency with our definitions of hip fracture and were interested in events that create substantial disruption for patients and families. We used prior algorithms to identify ICD-9 codes and cross-walked them to ICD-10 codes to define pneumonia (Methods S2) [23–25]. We included ICD codes in any position in the claim (principal or secondary) and applied a 30-day period to define a single pneumonia event.

#### Mortality:

We ascertained 1-year mortality using a reconciliation process to identify date of death from data available in the HRS, a linkage to the National Death Index, or Medicare claims. Virtually all deaths are captured with this approach [26].

#### Function:

The HRS core and exit surveys include items on whether participants had difficulty or required assistance with activities of daily living. As in prior studies [27], we defined functional impairment as requiring assistance with any of the 6 Activities of Daily Living (ADL; walking, dressing, bathing, eating, getting into and out of bed, and toileting) or 5 Instrumental Activities of Daily Living (IADLs; preparing a hot meal, shopping for groceries, making telephone call, taking medicines, and managing money). These responses are summed to create a total score of 0–11. To better illustrate functional decline, we reverse coded scores so that 11 indicates requiring no assistance and 0 indicates requiring assistance on all ADL’s and IADL’s.

In the HRS exit interview, which is administered for participants who die, proxies are asked to report on the participants function in the last 3 months of life. These questions are skipped if the proxy reports that the participant stayed in bed for more than 85 days prior to death. For these individuals, we assigned a score of 4 on the 11-item reverse-coded scale. Nursing home residents were also assigned a score of 4 since questions regarding IADL’s are skipped for nursing home residents. 15% of the hip fracture cohort and 12% of the pneumonia cohort had an imputed score. These decisions to assign a score of 4 were based on clinical grounds, since in our experience individuals who are bedbound or qualify for nursing home care at end of life have high levels of disability.

#### Dementia Status:

We used the previously validated Hurd algorithm to define dementia status [7]. This algorithm uses cognitive and functional data from HRS interviews to estimate a predicted probability of dementia at the time of each interview [7]. The Hurd algorithm has been shown to have a high specificity (93%) and accuracy (88%), albeit relatively low sensitivity (39%). Overall performance of the algorithm was high compared to other algorithms used to identify dementia in Health and Retirement Study data [28].

#### Other measures:

Patient sociodemographic and clinical characteristics used to describe the sample or for matching and adjustment included age, sex, education level, household wealth, enrollment in Medicare Part C, average number of comorbidities, and self-reported race and ethnicity. For self-reported race and ethnicity, we categorized participants as non-Hispanic White, non-Hispanic Black, or Hispanic. Due to small sample size, other race and ethnicity categories were not included.

### 2.3 | Statistical Analysis

For mortality, we used Cox regression to estimate predicted 1-year mortality and hazard ratios, with censoring at 1-year if death did not occur. We depicted results visually using Kaplan–Meier survival curves. To model function, we used a linear mixed effects regression model to estimate pre- and post-event annual slopes up to 2-waves or 5 years before/after the actual or synthetic events. For those who experienced an event, we used a linear spline with a knot at time 0 and included an indicator term for immediate change in function at time of event. All models were adjusted for age, sex, dementia status, number of comorbidities, and education level [29]. We present unweighted results for our primary findings since our sample included small population subgroups and weighted estimates may not be stable, although we do include weighted estimates in sensitivity analyses [30].

#### Sensitivity analyses:

We conducted a number of sensitivity analyses to confirm the robustness of our findings. For mortality, analyses were additionally adjusted for survey weights. For function, analyses included: (1) Poisson regression; (2) weighting for death and drop-out and/or survey-weighting; (3) time-varying covariate approach; (4) limiting analysis to individuals who had functional impairment (function score less than or equal to 9) at time of real or synthetic hip fracture or pneumonia; (5) Alternative imputations for missing function scores from exit interviews or for nursing home residents; (6) Pneumonia ICD code in first versus secondary position. We also created an alternative figure that used cubic splines instead of a linear model to depict function. More details on these sensitivity analyses and rationale are available in the appendix (Methods S3).

Analyses were conducted using SAS Version 9.4 (SAS Institute, Cary, North Carolina) and STATA Version 18 (StataCorp LLC, College Station, TX). This study was approved by the UCSF and ISMMS Internal Review Boards.

## 3 | Results

Characteristics of the hip fracture or pneumonia cohorts were similar after matching (Table 1). For example, among people with dementia, those with hip fracture had similar mean age (87.7) and proportion female (79%) as people with dementia with no hip fracture (mean age 87.1, 79% female).

### 3.1 | Comparison 1: People With Dementia With and Without Hospitalization for Hip Fracture or Pneumonia

#### 3.1.1 | Mortality

##### Hip Fracture:

**FIGURE 1 |:**
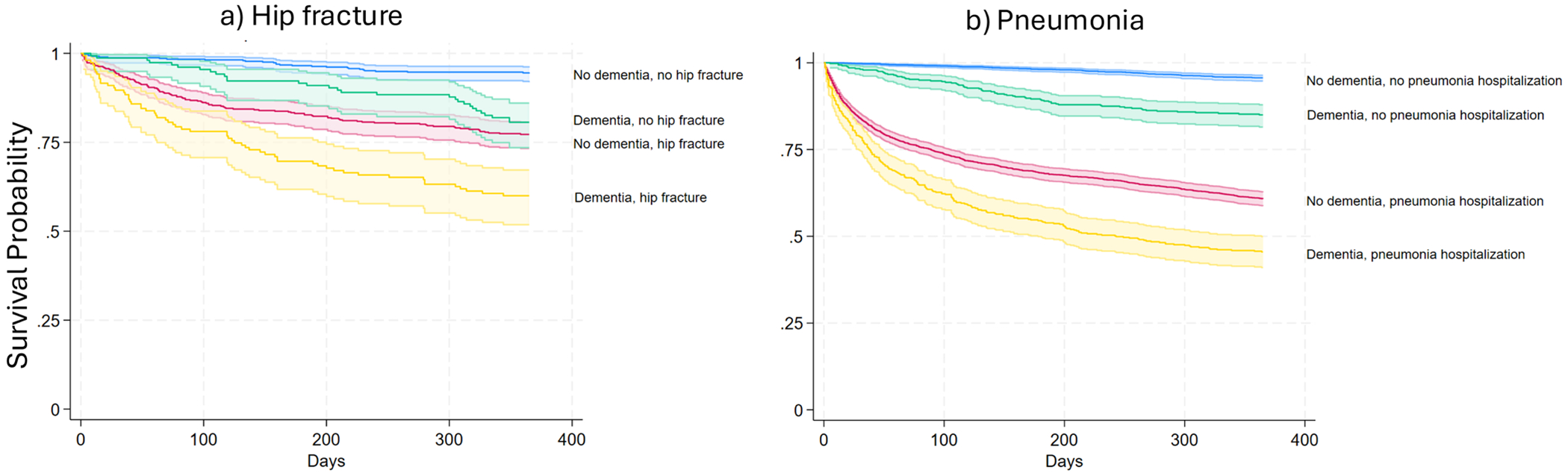
Kaplan–Meier survival curves for people with and without dementia and with and without hospitalization for (a) hip fracture or (b) pneumonia. Shaded lines indicate 95% Confidence Intervals.

People with dementia with hip fracture had a higher predicted 1-year mortality of 35.4% compared to 14.8% in people with dementia without hip fracture (Adjusted Hazard Ratio (AHR) 2.74, 95% Confidence Interval (CI) 1.75, 4.27) (Table 2; Figure 1a).

##### Pneumonia:

People with dementia with pneumonia had a higher predicted 1-year mortality of 49.6% compared to 13.0% for people with dementia without pneumonia (AHR 4.94, 95% CI 3.81, 6.42) (Table 2; Figure 1b).

#### 3.1.2 | Function

##### Hip Fracture:

**FIGURE 2 |:**
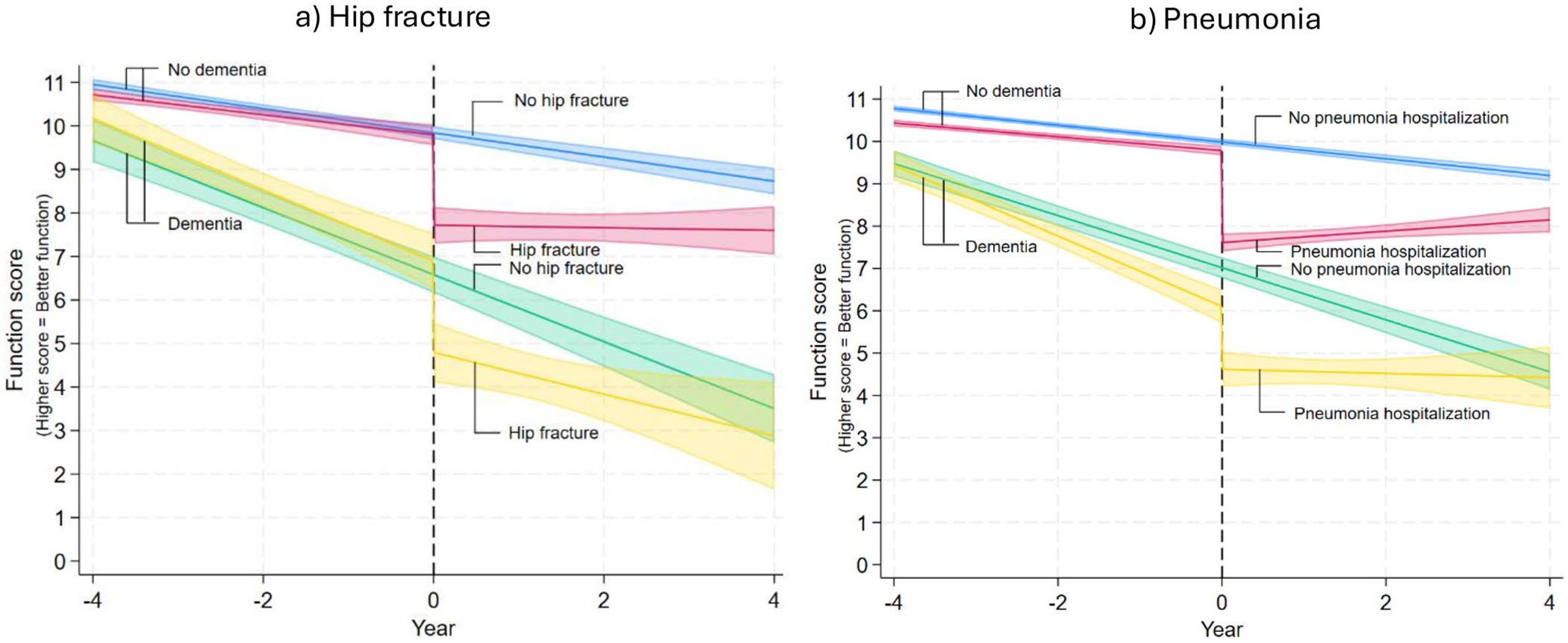
Predicted function score for people with and without dementia and with and without hospitalization for (a) hip fracture or (b) pneumonia. Function is defined on a reverse-coded 0–11 scale that is the sum of requiring assistance in 6 activities of daily living and 5 instrumental activities of daily living (higher scores indicate better function). Shaded lines indicate 95% Confidence Intervals. Year 0 indicates time of real or synthetic hip fracture or pneumonia.

Function scores of people with dementia with hip fracture were declining by −0.82/year (95% CI −1.05, −0.59) prior to hip fracture, dropped by −2.09 points (95% CI −2.94, −1.25) at time of hip fracture, and continued to decline by −0.48 points/year (95% CI −0.87, −0.09) after hip fracture. People with dementia without hip fracture declined by −0.77 points per year (95% CI −0.90, −0.64) over the trajectory (Table 3, Figure 2a).

##### Pneumonia:

Function scores of people with dementia with pneumonia were declining by −0.83 points/year (95% CI −0.97, −0.70) prior to pneumonia, dropped by 1.49 points at time of pneumonia hospitalization (95% CI −2.00, −0.97), and were flat after (−0.05 points/year [95% CI −0.29, 0.19]). People with dementia without pneumonia declined by −0.62 points per year (95% CI −0.68, −0.55) over the trajectory (Table 3, Figure 2b).

### 3.2 | Comparison 2: People With and Without Dementia With Hospitalization for Hip Fracture or Pneumonia

#### 3.2.1 | Mortality

##### Hip Fracture:

People with dementia with hip fracture had a higher predicted 1-year mortality of 35.4% compared to 24.0% in people without dementia with hip fracture (AHR 1.68 95% CI 1.20, 2.35) (Table 2; Figure 1a).

##### Pneumonia:

People with dementia with pneumonia had a higher predicted 1-year mortality of 49.6% compared to 39.7% in people without dementia with pneumonia (AHR 1.35, 95% CI 1.17, 1.57) (Table 2; Figure 1b).

#### 3.2.2 | Function

##### Hip Fracture:

People without dementia with hip fracture were declining by −0.23 points per year (95% CI −0.31, −0.15) prior to hip fracture, which was a significantly slower decline than for people without dementia with hip fracture (*p* ≤ 0.0001). They had a similar drop in function at time of hip fracture as compared to people with dementia with hip fracture (−2.08 points/year, 95% CI −2.51, −1.64, *p* = 0.1). After hip fracture, function for people without dementia with hip fracture stabilized (−0.03, 95% CI −0.22, 0.16) and was significantly different than slopes for people with dementia with hip fracture (*p* = 0.04) (Table 3, Figure 2a).

##### Pneumonia:

People without dementia with pneumonia were declining by −0.16 points per year (95% CI −0.20, −0.13) prior to event, which was a significantly slower decline than for people with dementia with pneumonia (*p* ≤ 0.0001) (Table 3). They had a slightly larger drop in function at time of pneumonia as compared to people with dementia with pneumonia (−2.17 points/year, 95% CI −2.39, −1.95, *p* = 0.02). After hospitalization for pneumonia, function for people without dementia with pneumonia increased (0.13 points/year, 95% CI 0.03, 0.24), but was not significantly different than slopes for people with dementia with hip fracture (*p* = 0.17).

##### Sensitivity analyses:

Results for all sensitivity analyses were similar (Tables S1–S4). One exception was a larger drop in function at time of hip fracture or pneumonia in the time-varying covariate model for both hip fracture and pneumonia analyses. This is to be expected given that some of the decline in the pre-event period for those with hip fracture or pneumonia would only be captured at time of event. The cubic spline model demonstrated similar trajectories to the linear model (Figure S2).

## 4 | Discussion

In this study using data from the population-based, longitudinal Health and Retirement Study with a community-based cohort, we found that both hip fracture and pneumonia are “disruptive” events for both people with and without dementia. These events, which occur more frequently in people with dementia [9], are also associated with worse outcomes for people with dementia. Compared to both people with dementia who do not experience these events or people without dementia who do experience events, people with dementia are more likely to die, experience profound immediate functional impairment and are less likely to recover function following both types of events. One third to one half of all people with dementia died within 1-year of experiencing hip fracture or pneumonia. Function scores following an event in people with dementia—which were already substantially lower in than those without dementia—dropped to a score of around 4 or below in activities of daily living (range 0–11), indicating at least several impairments in personal care needs such as toileting, transferring, or dressing. Our findings suggest that acute disruptive surgical and medical events such as hip fracture or pneumonia can profoundly alter both immediate and longer-term mortality and function and provide important prognostic information to clinicians, patients, and care partners [31–33].

Our study adds to a growing body of literature examining “disruptive events” or “health shocks” by focusing on a predominantly community-dwelling sample over a longer observation period than previous studies [34, 35]. Previous studies have either sampled from nursing home settings or hospital-based incident cohorts and lacked the comprehensive pre-event data available in HRS, lacked post-event follow-up after 6-months, or did not focus specifically on people with dementia [4, 8, 36, 37]. The expansion of findings to the community-dwelling dementia population is important given that two thirds of older adults with dementia live in non-institutional, community-based settings and federal and state payors continue to support policies that shift care from institutional settings to home and community-based settings [6]. Additionally, the longitudinal design of our study permits a more comprehensive understanding of both pre- and post-event trajectories. Previous studies have observed that disruptive events like hip fracture or pneumonia often follow a period of accelerated functional decline. Our study demonstrates this decline may be much more pronounced for people with dementia [20, 34, 38]. Our results also demonstrate that post-event trajectories for community-dwelling people with dementia are similar to nursing home residents with dementia, where previous work has shown that over 50% of residents with hip fracture or pneumonia died or experienced severe disability in the 6-months to 1-year following these events [2].

One notable finding was the high rates of mortality following hospitalization for pneumonia for both people with and without dementia. More than half of people with dementia who were hospitalized for pneumonia died within 1 year and 1-year mortality for people without dementia was higher than we observed in people with dementia with hip fracture. This high mortality rate may be because advances in oral antibiotic therapy means that many cases of community-acquired pneumonia no longer require hospitalization and as such, hospitalization is an indicator of very severe illness [39]. Future work should continue to examine the utility of hospitalization for pneumonia and subsequent intensity of care needed (e.g., intubation, intensive care unit admission, and duration of admission) as prognostic indicators.

Our findings have important implications for the clinical care of community-dwelling people with dementia who experience a disruptive event. Because of the insidious course of dementia, surgical and medical events such as hip fracture or pneumonia are often treated in isolation without consideration of the effect of dementia on outcomes [3, 4]. People with dementia and their families should be provided with information about the high likelihood of mortality and lack of functional recovery following these events. With few other accurate prognostic tools available for dementia [40], this information can help guide discussions about prognosis and goals of care conversation about treatment choices. Given knowledge of outcomes following these events, patients and families may opt for a conservative approach focused on comfort rather than intensive rehabilitation [41]. This may help patients and their families avoid pernicious cycling between skilled rehabilitation facilities and hospitalization in the final months of life, what some have termed “rehabbed to death” [42]. However, even people with dementia choosing a conservative approach to care could potentially benefit from a rehabilitation plan aimed at stabilization and preservation of function and improving quality of life, such as learning new strategies for transferring from bed to chair that would allow an individual to participate in communal meals [43].

This study used rigorous methods such as matching and multiple sensitivity analyses that address drawbacks of previous studies. The fact that multiple sensitivity analyses confirmed primary findings was reassuring. Several limitations to this study should be noted. First, we lack detail on dementia severity and subtype that might influence mortality and functional trajectories. However, our method to ascertain dementia relied on a validated algorithm that provide robust estimates at the population level. Second, although we both matched individuals with and without events on several demographic and clinical characteristics—and pre-event slopes indicated the groups were similar—it is possible that there is bias in our selection of controls that explains differences in post-event outcomes. However, a sensitivity analyses in which we limited analysis to individuals with functional impairment produced similar results. Finally, we selected hip fracture and pneumonia as highly relevant exemplars of disruptive surgical and medical events, and it is unknown if these findings generalize to other acute events such as a stroke or acute myocardial infarction [34]. Future research should explore whether similar patterns are found for different types of events, including both clinical and social (e.g., widowhood) events.

In conclusion, acute events including hospitalization for hip fracture or pneumonia, which already occur more frequently in community-dwelling people with dementia, are associated with higher mortality and functional impairment compared to people with dementia who do not experience these disruptive events and people without dementia who do experience events. This information can help guide discussions about prognosis, goals of care, and treatment choices when these events occur.

## Supplementary Material

supplementary materials

Additional supporting information can be found online in the Supporting Information section.

## Figures and Tables

**TABLE 1 |:** Characteristics of participants after matching.^a^

	No dementia		Dementia	
	No hip fracture (*n* = 493)	Hip fracture (*n* = 493)	No hip fracture (*n* = 155)	Hip fracture (*n* = 155)
Age, mean (SD)	82.4 (7.0)	82.5 (7.1)	87.1 (6.5)	87.7 (6.6)
Female, *n* (%)	357 (72%)	357 (72%)	122 (79%)	122 (79%)
Race and ethnicity^b^				
White	372 (75%)	438 (89%)	98 (63%)	120 (77%)
Black	77 (16%)	< 10%	< 25%	< 15%
Hispanic	36 (7%)	< 5%	< 5%	< 5%
High school or GED, *n* (%)	164 (33%)	170 (34%)	48 (31%)	53 (34%)
Median household total wealth (IQR), US $	205,050 (59,900, 521,000)	162,000 (47,000, 425,000)	50,025 (100, 177,000)	75,000 (4000, 40,400)
Married or partnered, *n* (%)	216 (44%)	197 (40%)	31 (20%)	40 (26%)
Lives alone, *n* (%)	185 (38%)	262 (53%)	79 (51%)	78 (50%)
Medicare Part C, *n* (%)	294 (60%)	340 (69%)	111 (72%)	111 (72%)
Comorbidities, mean (SD)^c^	1.9 (1.1)	2.0 (1.1)	2.0 (1.2)	1.7 (1.2)
Difficulty in 2 or more ADL’s, *n* (%)	49 (10%)	83 (17%)	68 (44%)	59 (38%)
Function score, 0–11 (higher score = better function), mean (SD)	10.5 (1.1)	10.3 (1.5)	7.4 (3.3)	7.6 (3.1)
	No pneumonia (*n* = 2305)	Pneumonia (*n* = 2305)	No pneumonia (*n* = 480)	Pneumonia (*n* = 480)

Age, mean (SD)	79.5 (7.0)	79.7 (7.4)	84.8 (7.6)	85.8 (7.9)
Female, *n* (%)	1154 (50%)	1154 (50%)	273 (57%)	273 (57%)
High school or GED, *n* (%)	777 (34%)	771 (33%)	119 (33%)	121 (25%)
Race and ethnicity^b^				
White	1782 (77%)	1813 (79%)	296 (62%)	305 (64%)
Black	303 (13%)	297 (13%)	93 (19%)	100 (21%)
Hispanic	165 (7%)	147 (6%)	78 (16%)	68 (14%)
Median household total wealth (IQR), US $	186,150 (50,000, 493,000)	142,000 (27,000, 403,000)	58,000 (950, 25,500)	70,500 (1225, 252,400)
Married or partnered, *n* (%)	1258 (55%)	1134 (49%)	174 (36%)	162 (34%)
Lives alone, *n* (%)	702 (30%)	719 (31%)	156 (34%)	149 (31%)
Medicare Part C, *n* (%)	1441 (63%)	1607 (70%)	303 (63%)	347 (72%)
Comorbidities, mean (SD)^c^	2.3 (1.1)	2.3 (1.2)	2.0 (1.1)	2.0 (1.3)
Difficulty in 2 or more ADL’s, *n* (%)	250 (11%)	441 (19%)	206 (43%)	257 (54%)
Function score, 0–11 (higher score = better function), mean (SD)	10.6 (1.1)	10.2 (1.7)	7.4 (3.4)	6.7 (3.5)

Abbreviations: ADL = Activity of Daily Living; GED = General equivalency diploma; IQR = Interquartile range; SD = standard deviation.

a Participants with an event (hip fracture or pneumonia) were matched to those without the event by first calculating propensity scores with the covariates age, sex, dementia status, number of comorbidities, and education level and then using propensity score matching, with exact matching required for age, sex, interview wave, and dementia status. For the event group, the date of the event was set as time 0. For those who did not have an event, we calculated a synthetic event time based on the interval start date and the matched case event time.

b Due to small sample sizes and cell size reporting restrictions, numbers for some cells of Black and Hispanic participants are reported as being less than a certain proportion rather than the exact number and proportion. Other racial and ethnic categories are not reported due to small sample size and thus percentages do not add to 100%.

c Comorbidities included heart disease, lung disease, stroke, cancer, arthritis, and diabetes.

**TABLE 2 |:** Association between hospitalization for hip fracture or pneumonia and predicted 1-year mortality for people with and without dementia.

	Predicted 1-year mortality^a^	Hazard ratio (95% confidence interval)		
Hip fracture				
No dementia/No hip fracture	5.5%			Reference
No dementia/Hip fracture	24.0%		Reference	4.64 (3.07, 7.02)
Dementia/No hip fracture	14.8%	Reference		2.85 (1.67, 4.87)
Dementia/Hip Fracture	35.4%	2.74 (1.75, 4.27)	1.68 (1.20, 2.35)	7.81 (4.91, 12.42)
Pneumonia				
No dementia/No pneumonia	4.3%			Reference
No dementia/Pneumonia	39.7%		Reference	11.58 (9.42, 14.24)
Dementia/No Pneumonia	13.0%	Reference		3.17 (2.34, 4.31)
Dementia/Pneumonia	49.6%	4.94 (3.81, 6.42)	1.35 (1.17, 1.57)	15.68 (12.37, 19.86)

a Mortality was modeled using Cox regression with censoring at 1 year if death did not occur. Participants with and without events were matched by calculating propensity scores with the covariates age, sex, dementia status, number of comorbidities, and education level and then using propensity score matching, with exact matching required for sex, survey wave, and dementia status. All models were additionally adjusted for age, sex, dementia status, number of comorbidities, and education level.

**TABLE 3 |:** Association between hospitalization for hip fracture or pneumonia and function for people with and without dementia.

	Function^a,b^		
	Pre-event slope (95% CI)	Drop at time zero (95% CI)	Post-event slope (95% CI)
Hip fracture			
No dementia/No hip fracture	−0.28 (−0.32, −0.23)		−0.28 (−0.32, −0.23)
No dementia/Hip fracture	−0.23 (−0.31, −0.15)	−2.08 (−2.51, −1.64)	−0.03 (−0.22, 0.16)
Dementia/No hip fracture	−0.77 (−0.90, −0.64)		−0.77 (−0.90, −0.64)
Dementia/Hip Fracture	−0.82 (−1.05, −0.59)	−2.09 (−2.94, −1.25)	−0.48 (−0.87, −0.09)
Pneumonia			
No dementia/No pneumonia	−0.20 (−0.21, −0.18)		−0.20 (−0.21, −0.18)
No dementia/Pneumonia	−0.16 (−0.20, −0.13)	−2.17 (−2.39, −1.95)	0.13 (0.03, 0.24)
Dementia/No Pneumonia	−0.62 (−0.68, −0.55)		−0.62 (−0.68, −0.55)
Dementia/Pneumonia	−0.83 (−0.97, −0.70)	−1.49 (−2.00, −0.97)	−0.05 (−0.29, 0.19)

Abbreviation: CI = Confidence Interval.

a Function was defined as the sum of the number of 6 activities of daily living and 5 instrumental activities of daily living in which the respondent did not require assistance (higher scores indicate better function).

b Function was modeled using linear spline model to estimate pre- and post-event annual slopes up to 2 waves or 5 years before/after the actual or synthetic event times with knots placed at time of event. Participants with and without events were matched by calculating propensity scores with the covariates age, sex, dementia status, number of comorbidities, and education level and then using propensity score matching, with exact matching required for sex, survey wave, and dementia status. Models were additionally adjusted for age, sex, dementia status, number of comorbidities, and education level.

## Summary

- Key points
  - “Disruptive” acute events occur more frequently among people with dementia than people without dementia and may accelerate decline. We compared the experience of hospitalization for hip fracture (a surgical event) or pneumonia (a medical event) among people with dementia to two groups: (1) people with dementia who did not experience these events; and (2) people without dementia who experienced an event.
  - Compared to people with dementia who did not experience these events, people with dementia hospitalized for either hip fracture or pneumonia had higher 1-year mortality and higher rates of functional decline.
  - Compared to people without dementia hospitalized for either hip fracture or pneumonia, people with dementia had higher 1-year mortality and lower rates of functional recovery.
- Why does this paper matter?
  - Disruptive events such as hip fracture or pneumonia substantially alter the clinical trajectories of people with dementia and are associated with worse outcomes than in people without dementia who experience these events.
  - This information can be used to improve anticipatory guidance and inform goal-concordant care for people with dementia.
