# Supplementary material for: Mortality and Function After Hip Fracture or Pneumonia in People With and Without Dementia
Source: J Am Geriatr Soc. Author manuscript; Available in PMC 2026 Mar 12. (PMC12981159; doi:10.1111/jgs.19354)
Supplement: supplementary materials [file NIHMS2147431-supplement-supplementary_materials.pdf]

Table of Contents

Supplementary Methods S1. ICD-9 and ICD-10 codes used to define hip fracture 2

Supplementary Methods S2. ICD-9 and ICD-10 codes used to define pneumonia 3

Supplementary Methods S3. Additional detail on sensitivity analyses/alternative approaches 5

Supplementary Figure S1. Matching Approach 7

Supplementary Table S1. Sensitivity analyses for mortality outcome with hip fracture 8

Supplementary Table S2. Sensitivity analyses for mortality outcome with pneumonia 9

Supplementary Table S3. Sensitivity analyses for function outcome and hip fracture 10

Supplementary Table S4. Sensitivity analyses for function outcome and pneumonia 11

Supplementary Figure S2. Predicted function score for people with and without dementia and with and without hospitalization for a) hip fracture or b) pneumonia using cubic splines 12

References 13

Supplementary Methods S1. ICD-9 and ICD-10 codes used to define hip fracture

ICD-9 codes: included admissions with code 820.xx and excluded admissions that were considered late effects from a prior hip fracture (733.81, 733.82, 905.3, V540-V549). Diagnostic code in any position in the claim.

ICD-10 codes: S72.0XX, S72.1XX, M80.05X, M84.45, M80.85X, M84.65X with letters A, B, or C to indicate an initial encounter. Diagnostic code required to be in the first or first secondary position.[1,2]

Supplementary Methods S2. ICD-9 and ICD-10 codes used to define pneumonia

	ICD-9	ICD-10
Viral pneumonia	480.x	J12.xx
Pneumonia due to adenovirus	480.0	J12.xx
Pneumonia due to respiratory syncytial virus	480.1	J12.xx
Pneumonia due to parainfluenza virus	480.2	J12.xx
Pneumonia due to other virus not classified otherwise	480.8	J12.xx
Viral pneumonia	480.9	J12.xx
Pneumococcal pneumonia	481	J13
Other bacterial pneumonia	482.xx	J15.xx
Pneumonia due to Klebsiella pneumonia	482.0	J15.xx
Pneumonia due to pseudomonas	482.1	J15.xx
Pneumonia due to Haemophilus influenzae	482.2	J15.xx
Pneumonia due to Streptococcus	482.3x	J15.xx
Pneumonia due to staphylococcus	482.4x	J15.xx
Pneumonia due to other specified bacteria	482.8x	J15.xx
Bacterial pneumonia unspecified	482.9	J15.xx
Pneumonia due to other specified organism	483.x	J16.xx
Pneumonia due to mycoplasma pneumoniae	483.0	J15.7
Pneumonia due to chlamydia	483.1	J16.0
Pneumonia due to other specified organism	483.8	J16.8
Bronchopneumonia and pneumonia, organism unspecified	485	J18.x
Bronchopneumonia and pneumonia, organism unspecified	486	J18.x
Pneumonia in infectious disease classified elsewhere	484	Not applicable
Pneumonia in cytomegalic inclusion disease	484.1	B25.0
Pneumonia in whooping cough	484.3	A37.91
Pneumonia in anthrax	484.5	A22.1
Pneumonia in aspergeillosis	484.6	B44.0
Pneumonia in other systemic mycoses	484.7	J17
Pneumonia in other infectious disease classified elsewhere	484.8	J17
Influenza with pneumonia	487.0	J11.00

Ornithoses with pneumonia	73.0	A70 with J17
Pulmonary actinomycosis	39.1	A42.0
Post-measles pneumonia	55.1	B05.2
Salmonella pneumonia	003.22	A02.22
Pneumonia due to toxoplasmosis	130.4	B58.3
Pulmonary tularemia	21.2	A21.2
Varicella pneumonia	52.1	B01.2
Empyema with fistula	510.0	J86.0
Empyema without fistula	510.9	J86.9
Pleurisy with effusion, bacterial cause other than tuberculosis	511.11	J90
Abscess of lung	513.0	J85.1
Pneumocytosis	136.3	B59
Candidiasis of the lung	112.4	B37.1
Primary coccidiodomycosis	114.0	B38.0
Histoplasmosis capsalatum pneumonia	115.05	B39.2
Histoplasmosis duboisii pneumonia	115.15	B39.5 with J17
Histoplasmosis unspecified pneumonia	115.95	B39.9 with J17
Aspiration pneumonia	507.0	J69.0

Lists drawn from Lave et al, Brereton et al, and Zhang et al.[3-5]

Supplementary Methods S3. Additional detail on sensitivity analyses/alternative approaches

Mortality:

1) Survey-weighting: To account for Health and Retirement complex survey design and unequal probability of selection. We conducted this analysis based on standard practice recommendations to compare both weighted and unweighted estimates. However, weighted estimates are generally not as impactful for multiple regression analyses and may not be as relevant in analyses of complex subgroups of the original survey.[6]

Function:

1) Poisson regression: To account for the possibility that function might better estimated as an integer with a score between 0 and 11 rather than as a continuous variable, we used Poisson regression instead of linear regression.
2) Weighting for death and drop out and/or survey-weighting
    a. Weighting for death and drop-out: To account for missing outcome data due to death and drop-out, we estimated models weighted by the inverse probability of survival (including non-dropout). We calculated survival probability at each interview by using a multivariable Cox model with the outcome as missing interviews within 2-waves after time 0 due to death or dropout. For subjects who had exit interview available 2-waves after time 0, we did not count them as died/dropped out.
    b. Survey-weighting:
        i. We first used Stata svyset command and svy prefix to conduct weighted analysis using HRS complex survey design features, including primary sampling unit (PSU), strata and sampling weights
        ii. We then used a 2-way cluster approach: In our study cohort, a case could also be included as a control before event. In the unweighted analysis presented as main findings, we used clustered robust standard error estimator with clusters for each subject to account for the correlation within subjects. However, robust standard error estimator is not supported in svy prefix. To account for correlation within subjects due to same subject can be both case and control, we created a cluster variable using PSU and strata for each subject and re-ran mix-effect linear regression with sampling weight and clustered robust standard error estimator with cluster created from PSU and strata as well as cluster for subject. Weighted results from these two methods were very similar.
    c. Weighting for death and drop out and survey sampling: We created a combined weight by multiplying weights constructed analysis 3 and 4.
3) Time-varying covariate approach: As an alternative to the matching approach used for the primary analysis described previously and used in all other sensitivity analyses, we constructed a model that included all waves of interviews for participants in the matched cohort. Hip fracture or pneumonia could occur as time-varying events. Dementia was treated a time-invariant covariate based on status at index interview. The cohort included

only individuals in the matched analysis approach. We opted not to use this approach for our primary analysis because there are concerns that estimates might be biased.[7]

4) Restricting analysis to individuals with function scores ≤ 9: To address concerns that the degree of functional impairment prior to hip fracture or pneumonia, particularly in comparing those with and without event, may influence the potential of impact of hip fracture or pneumonia on functional outcomes, limiting the ability to make causal inferences. To address this issue, we restricted analyses to include individuals who had functional impairment at baseline (i.e. time of event), defined as a function score or 9 or less, i.e. having at least 2 ADL or IADL impairments. This required conducting the matching approach with new cases and controls. For hip fracture, there were 168 cases matched to 162 controls and for pneumonia there were 713 cases matched to 708 controls.
5) Alternative approach for missing function scores: In the HRS exit interview, which is administered for participants who die, proxies are asked to report on the participants function in the last 3 months of life. These questions are skipped if the proxy reports that the participant stayed in bed for more than 85 days prior to death. For these individuals, we assigned a score of 4 on the 11-item reverse-coded scale. Nursing home residents were also assigned a score of 4 since questions regarding IADL's are skipped for nursing home residents. 15% of the hip fracture cohort and 12% of the pneumonia cohort had an imputed score. As a sensitivity analysis, we imputed alternative values for those with missing function scores: a) assigning a score of 3 or b) assigning a score of 5.
6) ICD code position for pneumonia: In our main analysis, pneumonia ICD code could be either the primary or secondary position. To address concerns that function outcomes might differ by diagnosis position, we conducted a sensitivity analysis where we stratified function outcomes by whether the pneumonia was in the a) primary (n=983 cases, 983 controls) or b) secondary position (n=1802 cases, 1802 controls).
7) Cubic spline model for function figure: As an alternative to depicting function with a linear spline model, we used a cubic spline model with 7 knots at -4 year, -1 year, -0.25 year, time 0, +0.25 year, +1 year, +4 years for those with an event and knots at 5, 27.5, 50, 72.5, and 95 percentiles for the non-event groups based on previous recommendations.[8]

Supplementary Figure S1. Matching Approach

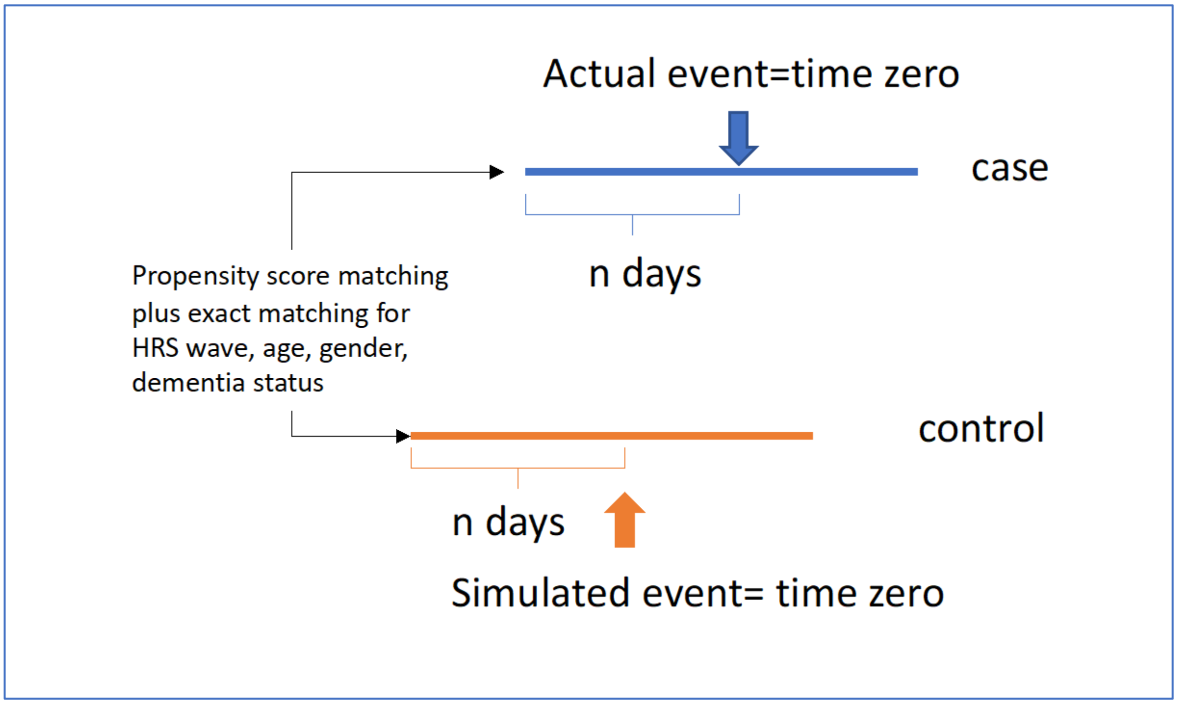

Within the analytic cohort, we identified cases of hip fracture and pneumonia among unique participants. We then matched participants with an event (case) to those without the event (control) at each wave by first calculating propensity scores with the covariates age, gender, dementia status, number of comorbidities, and education level and then using propensity score matching, with exact matching required for age, gender, HRS wave, and dementia status. For the event group, the date of the event was set as time zero. For those who did not have an event, we calculated a synthetic event time based on the interval start date and the matched case event time. This approach is recommended as a way to emulate clinical trials and enhance causal inference in observational studies.[9,10]

Supplementary Table S1. Sensitivity analyses for mortality outcome with hip fracture

	no dementia, no hip fracture			no dementia, yes hip fracture			yes dementia, no hip fracture			yes dementia, yes hip fracture		
	HR (95% CI)	1-year mortality	1-year death raw%	HR (95% CI)	1-year mortality	1-year death raw %	HR (95% CI)	1-year mortality	1-year death raw %	HR (95% CI)	1-year mortality	1-year death raw %
Unadjusted	Ref.	5.67%	28 (6%)	4.49 (2.97, 6.79)	23.04%	113 (23%)	3.55 (2.14, 5.88)	18.70%	30 (19%)	8.84 (5.66, 13.80)	40.30%	62 (40%)
Adjusted	Ref.	5.45%		4.64 (3.07, 7.02)	22.90%		2.85 (1.67, 4.87)	14.77%		7.81 (4.91, 12.42)	35.43%	
Survey weighted	Ref.	5.92%		4.13 (2.84, 6.01)	22.30%		3.26 (1.99, 5.35)	18.07%		8.55 (5.56, 13.13)	40.65%	

CI=Confidence interval; HR=Hazard ratio

Supplementary Table S2. Sensitivity analyses for mortality outcome with pneumonia

	no dementia, no pneumonia hospitalization			no dementia, yes pneumonia hospitalization			yes dementia, no pneumonia hospitalization			yes dementia, yes pneumonia hospitalization		
	HR (95% CI)	1-year mortality	1-year death raw %	HR (95% CI)	1-year mortality	1-year death raw %	HR (95% CI)	1-year mortality	1-year death raw %	HR (95% CI)	1-year mortality	1-year death raw %
Unadjusted	Ref.	4.30%	100 (4%)	11.48 (9.37, 14.08)	39.66%	911 (40%)	3.63 (2.70, 4.89)	14.77%	72 (15%)	18.23 (14.51, 22.90)	55.16%	263 (55%)
Adjusted	Ref.	4.28%		11.58 (9.42, 14.24)	39.74%		3.17 (2.34, 4.31)	12.95%		15.68 (12.37, 19.86)	49.62%	
Survey weighted	Ref.	3.99%		12.02 (9.43, 15.33)	38.68%		3.87 (2.75, 5.44)	14.56%		19.39 (14.94, 25.17)	54.57%	

CI=Confidence interval; HR=Hazard ratio

Supplementary Table S3. Sensitivity analyses for function outcome and hip fracture

	no dementia, no hip fracture			no dementia, yes hip fracture			yes dementia, no hip fracture			yes dementia, yes hip fracture		
	pre-slope	post-slope	drop	pre-slope	post-slope	drop	pre-slope	post-slope	drop	pre-slope	post-slope	drop
1:Poisson regression	-0.20 (-0.26, -0.15)		--	-0.11 (-0.15, 0.07)	-0.23 (-0.41, -0.06)	-0.86 (-1.12, -0.60)	-0.54 (-0.71, -0.38)		--	-0.48 (-0.63, -0.33)	-0.60 (-1.04, -0.16)	-0.63 (-1.31, 0.05)
2a: Inverse Probability Weighted	-0.28 (-0.33, -0.23)		--	-0.24 (-0.32, -0.16)	-0.05 (-0.23, 0.13)	-2.01 (-2.45, -1.57)	-0.77 (-0.90, -0.64)		--	-0.83 (-1.06, -0.59)	-0.47 (-0.85, -0.09)	-2.10 (-2.95, -1.24)
2bi: Survey weighted svy	-0.29 (-0.34, -0.23)		--	-0.20 (-0.27, -0.13)	-0.07 (-0.26, 0.12)	-2.01 (-2.44, -1.58)	-0.78 (-0.90, -0.66)		--	-0.81 (-1.02, -0.61)	-0.31 (-0.72, 0.09)	-2.47 (-3.27, -1.68)
2bii:Survey weighted cluster	-0.29 (-0.34, -0.24)		--	-0.20 (-0.26, -0.13)	-0.07 (-0.26, 0.13)	-2.01 (-2.44, -1.58)	-0.79 (-0.91, -0.66)		--	-0.81 (-1.03, -0.60)	-0.31 (-0.77, 0.15)	-2.47 (-3.33, -1.62)
2c: IPW + Survey weighted	-0.29 (-0.35, -0.23)		--	-0.21 (-0.28, -0.13)	-0.09 (-0.27, 0.09)	-1.95 (-2.37, -1.52)	-0.78 (-0.91, -0.66)		--	-0.83 (-1.05, -0.62)	-0.31 (-0.72, 0.09)	-2.45 (-3.30, -1.60)
3: Time-varying covariates	-0.11 (-0.14, -0.09)		--	--	-0.07 (-0.14, 0.01)	-2.37 (-2.64, -2.10)	-0.38 (-0.42, -0.34)		--	--	-0.07 (-0.26, 0.12)	-4.97 (-5.50, -4.45)
4. Function scores $\leq$ 9:	-0.48 (-0.63, -0.33)		--	-1.05 (-1.30, -0.79)	0.18 (-0.27, 0.63)	-1.66 (-2.80, -0.51)	-0.80 (-0.97, -0.62)		--	-1.20 (-1.50, -0.90)	-0.38 (-0.77, 0.003)	-1.06 (-2.03, -0.09)
5a: Imputed missing function=3	-0.29 (-0.34, -0.24)		--	-0.23 (-0.31, -0.15)	-0.01 (-0.21, 0.18)	-2.21 (-2.67, -1.75)	-0.80 (-0.93, -0.67)		--	-0.82 (-1.05, -0.59)	-0.41 (-0.81, -0.02)	-2.48 (-3.35, -1.62)
5b: Imputed missing function=5	-0.27 (-0.31, -0.22)		--	-0.23 (-0.30, -0.15)	-0.05 (-0.22, 0.13)	-1.94 (-2.36, -1.53)	-0.74 (-0.86, -0.61)		--	-0.82 (-1.05, -0.59)	-0.55 (-0.94, -0.15)	-1.71 (-2.59, -0.84)

Supplementary Table S4. Sensitivity analyses for function outcome and pneumonia

	no dementia, no pneumonia			no dementia, yes pneumonia			yes dementia, no pneumonia			yes dementia, yes pneumonia		
	pre-slope	post-slope	drop	pre-slope	post-slope	drop	pre-slope	post-slope	drop	pre-slope	post-slope	drop
1:Poisson regression	-0.10 (-0.11, -0.08)		--	-0.07 (-0.08, -0.06)	-0.17 (-0.26, 0.08)	-0.79 (-0.91, -0.67)	-0.38 (-0.46, -0.31)		--	-0.42 (-0.49, -0.35)	-0.14 (-0.35, 0.08)	-0.43 (-0.80, -0.07))
2a: IPW	-0.20 (-0.22, -0.18)		--	-0.17 (-0.20, -0.13)	0.11 (0.01, 0.21)	-2.12 (-2.33, -1.90)	-0.61 (-0.68, -0.54)		--	-0.86 (-1.00, -0.72)	-0.07 (-0.32, 0.17)	-1.41 (-1.94, -0.89)
2bi: Survey weighted svy	-0.19 (-0.21, -0.17)		--	-0.13 (-0.16, -0.10)	0.18 (0.07, 0.29)	-2.21 (-2.48, -1.94)	-0.65 (-0.73, -0.57)		--	-0.80 (-0.97, -0.63)	-0.03 (-0.27, 0.22)	-1.69 (-2.29, -1.08)
2bii:Survey weighted cluster	-0.19 (-0.21, -0.17)		--	-0.13 (-0.16, -0.10)	0.18 (0.07, 0.29)	-2.21 (-2.46, -1.96)	-0.65 (-0.73, -0.56)		--	-0.80 (-0.97, -0.62)	-0.03 (-0.30, 0.24)	-1.69 (-2.33, -1.04)
2c: IPW + Survey weighted	-0.19 (-0.22, -0.17)		--	-0.13 (-0.16, -0.10)	0.15 (0.05, 0.26)	-2.16 (-2.43, -1.88)	-0.64 (-0.72, -0.56)		--	-0.83 (-1.01, -0.65)	-0.06 (-0.30, 0.18)	-1.61 (-2.22, -0.99)
3: Time-varying covariates	-0.14 (-0.16, -0.12)		--	--	-0.07 (-0.14, 0.01)	-2.57 (-2.84, -2.31)	-0.43 (-0.47, -0.39)		--	--	-0.08 (-0.26, 0.11)	-5.30 (-5.81, -4.78)
4. function scores ≤ 9:	-0.46 (-0.53, -0.39)			-0.74 (-0.85, -0.63)	0.02 (-0.26, 0.30)	-1.14 (-1.67, -0.62)	-0.77 (-0.85, -0.68)			-1.16 (-1.33, -1.00)	-0.06 (-0.32, 0.21)	-0.32 (-0.85, 0.21)
5a: Imputed missing function=3	-0.21 (-0.23, -0.19)			-0.16 (-0.20, -0.13)	0.17 (0.06, 0.27)	-2.35 (-2.58, -2.12)	-0.64 (-0.71, -0.57)			-0.83 (-0.96, -0.70)	-0.01 (-0.26, 0.24)	-1.83 (-2.36, -1.31)
5b: Imputed missing function=5	-0.19 (-0.21, -0.17)			-0.16 (-0.19, -0.13)	0.10 (0.003, 0.20)	-2.00 (-2.21, -1.79)	-0.59 (-0.66, -0.52)			-0.83 (-0.97, -0.70)	-0.09 (-0.33, 0.15)	-1.14 (-1.65, -0.63)
6a: Primary ICD position	-0.21 (-0.24, -0.17)			-0.15 (-0.20, -0.10)	0.07 (-0.09, 0.23)	-1.97 (-2.32, -1.63)	-0.67 (-0.77, -0.57)			-0.90 (-1.11, -0.70)	-0.11 (-0.45, 0.23)	-0.93 (-1.63, -0.23)
6b: Secondary ICD position	-0.19 (-0.22, -0.17)			-0.17 (-0.21, -0.13)	0.17 (0.04, 0.31)	-2.31 (-2.59, -2.02)	-0.58 (-0.67, -0.49)			-0.79 (-0.96, -0.62)	0.001 (-0.34, 0.34)	-1.90 (-2.62, -1.17)

Supplementary Figure S2. Predicted function score for people with and without dementia and with and without hospitalization for a) hip fracture or b) pneumonia using cubic splines

a) Hip fracture

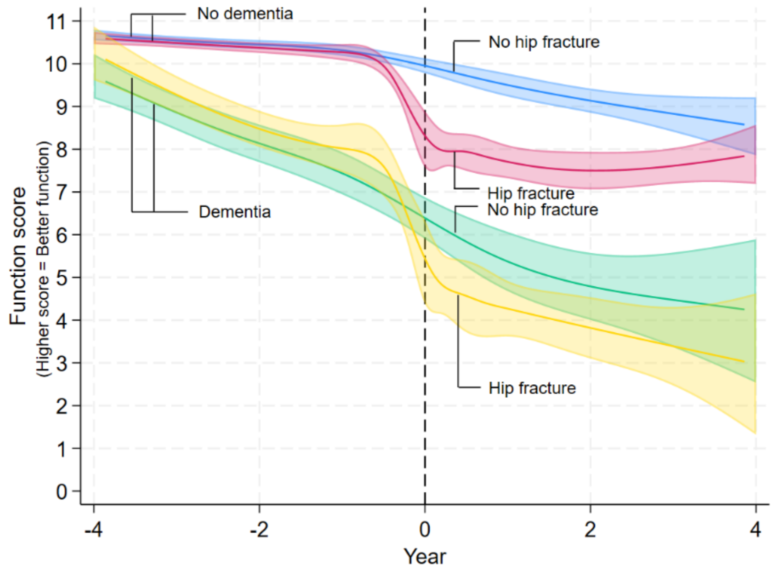

a) Pneumonia

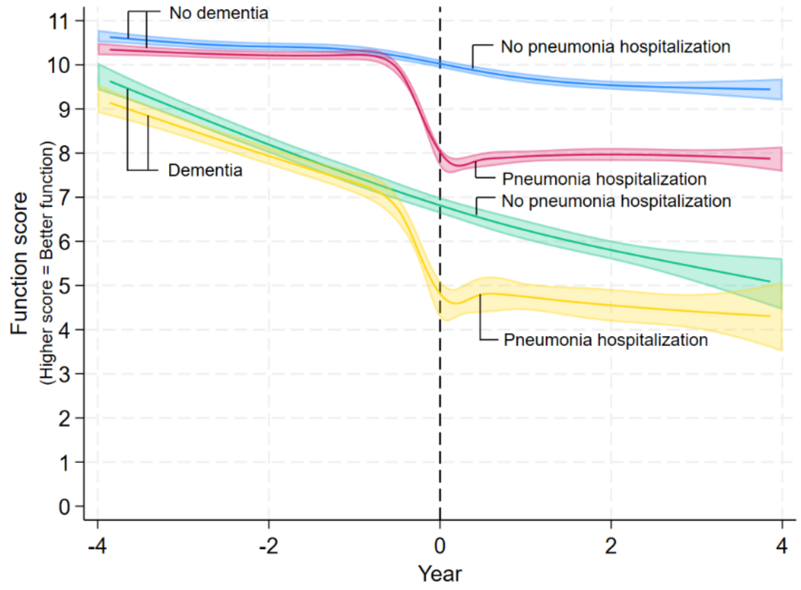

References

1. Rigler SK, Ellerbeck E, Whittle J, Mahnken J, Cook-Wiens G, Shireman TI. Comparing methods to identify hip fracture in a nursing home population using Medicare claims. *Osteoporosis international : a journal established as result of cooperation between the European Foundation for Osteoporosis and the National Osteoporosis Foundation of the USA*. Jan 2011;22(1):57-61. doi:10.1007/s00198-010-1264-8
2. Berry SD, Zullo AR, McConeghy K, Lee Y, Daiello L, Kiel DP. Defining hip fracture with claims data: outpatient and provider claims matter. *Osteoporosis international : a journal established as result of cooperation between the European Foundation for Osteoporosis and the National Osteoporosis Foundation of the USA*. Jul 2017;28(7):2233-2237. doi:10.1007/s00198-017-4008-1
3. Lave JR, Fine MJ, Sankey SS, Hanusa BH, Weissfeld LA, Kapoor WN. Hospitalized pneumonia. Outcomes, treatment patterns, and costs in urban and rural areas. *J Gen Intern Med*. Jul 1996;11(7):415-21. doi:10.1007/BF02600189
4. Brereton CJ, Lennon D, Browning S, Dunn E, Ferguson JK, Davis JS. Is gentamicin safe and effective for severe community-acquired pneumonia? An 8-year retrospective cohort study. *Int J Antimicrob Agents*. Jun 2018;51(6):862-866. doi:10.1016/j.ijantimicag.2018.01.018
5. Zhang D, Petigara T, Yang X. Clinical and economic burden of pneumococcal disease in US adults aged 19-64 years with chronic or immunocompromising diseases: an observational database study. *BMC Infect Dis*. Aug 29 2018;18(1):436. doi:10.1186/s12879-018-3326-z
6. Si Y, Lee S, Heeringa SG. Population Weighting in Statistical Analysis. *JAMA internal medicine*. Jan 1 2024;184(1):98-99. doi:10.1001/jamainternmed.2023.6300
7. Austin PC, Latouche A, Fine JP. A review of the use of time-varying covariates in the Fine-Gray subdistribution hazard competing risk regression model. *Stat Med*. Jan 30 2020;39(2):103-113. doi:10.1002/sim.8399
8. Harrell FE. *Regression Modeling Strategies: With Applications to Linear Models, Logistic Regression, and Survival Analysis.* Springer; 2001.
9. Hernán MA, Wang W, Leaf DE. Target Trial Emulation: A Framework for Causal Inference From Observational Data. *JAMA : the journal of the American Medical Association*. Dec 27 2022;328(24):2446-2447. doi:10.1001/jama.2022.21383
10. Ioannou GN, Bohnert ASB, O'Hare AM, et al. Effectiveness of mRNA COVID-19 Vaccine Boosters Against Infection, Hospitalization, and Death: A Target Trial Emulation in the Omicron (B.1.1.529) Variant Era. *Annals of internal medicine*. Dec 2022;175(12):1693-1706. doi:10.7326/m22-1856
